# 3D modeling and printing for complex biventricular repair of double outlet right ventricle

**DOI:** 10.3389/fcvm.2022.1024053

**Published:** 2022-11-30

**Authors:** Jan Brüning, Peter Kramer, Leonid Goubergrits, Antonia Schulz, Peter Murin, Natalia Solowjowa, Titus Kuehne, Felix Berger, Joachim Photiadis, Viktoria Heide-Marie Weixler

**Affiliations:** ^1^Institute for Cardiovascular Computer-Assisted Medicine, Charité – Universitätsmedizin Berlin, Berlin, Germany; ^2^Partner Site Berlin, German Center for Cardiovascular Research (DZHK), Berlin, Germany; ^3^Department of Congenital Heart Disease/Pediatric Cardiology, German Heart Center Berlin, Berlin, Germany; ^4^Einstein Center Digital Future, Berlin, Germany; ^5^Department of Congenital Heart Surgery and Pediatric Heart Surgery, German Heart Center Berlin, Berlin, Germany; ^6^Berlin Institute of Health (BIH), Berlin, Germany; ^7^Department of Cardiothoracic and Vascular Surgery, German Heart Center Berlin, Berlin, Germany

**Keywords:** double outlet right ventricle (DORV), biventricular repair, 3D printing, congenital heart disease, image reconstruction

## Abstract

**Background:**

Double outlet right ventricle (DORV) describes a group of congenital heart defects where pulmonary artery and aorta originate completely or predominantly from the right ventricle. The individual anatomy of DORV patients varies widely with multiple subtypes classified. Although the majority of morphologies is suitable for biventricular repair (BVR), complex DORV anatomy can render univentricular palliation (UVP) the only option. Thus, patient-specific decision-making is critical for optimal surgical treatment planning. The evolution of image processing and rapid prototyping techniques facilitate the generation of detailed virtual and physical 3D models of the patient-specific anatomy which can support this important decision process within the Heart Team.

**Materilas and methods:**

The individual cardiovascular anatomy of nine patients with complex DORV, in whom surgical decision-making was not straightforward, was reconstructed from either computed tomography or magnetic resonance imaging data. 3D reconstructions were used to characterize the morphologic details of DORV, such as size and location of the ventricular septal defect (VSD), atrioventricular valve size, ventricular volumes, relationship between the great arteries and their spatial relation to the VSD, outflow tract obstructions, coronary artery anatomy, etc. Additionally, physical models were generated. Virtual and physical models were used in the preoperative assessment to determine surgical treatment strategy, either BVR vs. UVP.

**Results:**

Median age at operation was 13.2 months (IQR: 9.6-24.0). The DORV transposition subtype was present in six patients, three patients had a DORV-ventricular septal defect subtype. Patient-specific reconstruction was feasible for all patients despite heterogeneous image quality. Complex BVR was feasible in 5/9 patients (55%). Reasons for unsuitability for BVR were AV valve chordae interfering with potential intraventricular baffle creation, ventricular hypoplasia and non-committed VSD morphology. Evaluation in particular of qualitative data from 3D models was considered to support comprehension of complex anatomy.

**Conclusion:**

Image-based 3D reconstruction of patient-specific intracardiac anatomy provides valuable additional information supporting decision-making processes and surgical planning in complex cardiac malformations. Further prospective studies are required to fully appreciate the benefits of 3D technology.

## Introduction

Double outlet right ventricle (DORV) is a group of complex cardiac lesions, occurring in about 3-9/100,000 live births and is characterized by a malposition of the great arteries (aorta and pulmonary artery), which are both arising predominantly (>50%) or entirely from the right ventricle (RV) (1). There is a wide range of anatomic variation from a rather simple morphology of a ventricular septal defect (VSD) with overriding aorta to a complex one with transposition of the great arteries (TGA), outflow tract obstruction and a VSD. This certainly creates a heterogeneity in definition, classification and also surgical treatment options. Early classifications focused on morphological criteria based on the relationship between the VSD and the great arteries: 1. sub-aortic type, 2. sub-pulmonary type, 3. double committed type and 4. non-committed type (2). A more recent classification, adopted by the databases of the Society of Thoracic Surgeons and European Association of Cardiothoracic Surgery, classifies 4 subtypes of DORV according to their clinical aspect: 1. VSD-type, 2. Fallot-type, 3. TGA-type and 4. non-committed/remote VSD-type. This simplified classification, besides the location of the VSD, also considers the degree of right ventricular outflow tract obstruction (RVOTO). Based on these two criteria, together with ventricular dimensions, the degree of arterial malposition and possible additional cardiac malformations, surgical strategy has to be adapted. In complex cases, a careful individualized decision has to be made between feasibility of biventricular repair (BVR) or opting for univentricular palliation (UVP).

The non-physiological hemodynamics resulting from UVP may cause severe long-term complications and long-term outcome is therefore thought to be less favorable. Hence, BVR is the preferred approach (3). Current overall operative mortality rates for BVR in DORV patients of 6-7% are reported, however, the surgical feasibility and success rates are highly dependent on the morphological complexity (4, 5). Poorer outcome of BVR in patients with borderline ventricular hypoplasia or complex remote VSD morphology highlights the importance of careful preoperative assessment and surgical strategy planning (6). In general, ventricular hypoplasia, atrioventricular (AV) valve abnormalities, or unresolvable outflow tract obstruction are potential contraindications for BVR (7). In the non-committed or remote VSD-type, closure of the VSD to either artery may create a long intracavitary baffle with an acute angle resulting in high resistances and velocities and thus relevant pressure gradients or a potential baffle geometry that may cross the AV-valve inlet. Echocardiography is currently considered the reference standard in assessing cardiac anatomy and determining the preferable surgical approach in DORV patients (7). However, in patients, in whom surgical decision-making cannot be conclusively established based on echocardiography, new technologies such as 3D imaging can provide valuable additional information.

3D imaging techniques such as magnetic resonance imaging (MRI) (8) and computed tomography (CT) (9) are used increasingly frequent to visualize and analyze the anatomy of different complex congenital heart defects. These techniques allow visualization of the entire cardiovascular anatomy as well as measurements of relevant intracardiac dimensions and volumes (10). However, the representation of the 3D anatomy using two-dimensional (2D) slices perpendicular to each other, encumbers the assessment of the complex 3D structures and their position and orientation in relation to each other. These obstacles can be overcome by reconstruction of the 3D geometry of the patient-specific anatomy by means of image processing. This reconstruction can be performed using either CT or MRI data (11, 12). While several approaches and software packages exist to reconstruct the 3D surface geometry, manual interaction is usually required due to the complex anatomy and imaging artifacts. The resulting 3D reconstructions can then be visualized to better understand the complex anatomy.

In addition to approaches targeting visualization of the patient-specific 3D anatomy, physical models of it can be generated via 3D printing (13–15). These 3D printed models were found to allow better assessment of the spatial orientation of relevant intracardiac structures (11, 16). First studies already demonstrated that 3D printing prior to surgery improved understanding and surgical outcome parameters such as mechanical ventilation time or duration of intensive care unit (ICU) stay (17). Thus, 3D reconstruction of the patient-specific anatomy, especially in combination with 3D printing, might enhance understanding of the anatomical features and therefore provide a viable solution for the demand of precise preoperative planning and decision-making, that arises from the complexity of congenital heart defects.

This retrospective study addresses two aspects: First, by summarizing our experience over the last five years, the feasibility of 3D image reconstruction and 3D printing from preoperative imaging data is assessed and their additional value compared to echocardiography for surgical treatment planning of complex DORV patients, in whom decision-making is inconclusive, is appraised. Second, anatomical measurements, such as the size of the VSD and the heart valves’ annuli, as well as the ventricular volumes are compared between routine echocardiographic measurements and measurements performed on the 3D reconstructions. This comparison aims to address the question, whether 3D reconstructions simply provide additional information on the complex anatomy and the position of the intracardiac structures with respect to each other, or whether relevant measurements can also be performed directly using these reconstructions.

## Materials and methods

### Patient cohort

After institutional review board approval (EA2/116/22), informed consent was waived for retrospective data analysis. All DORV patients, operated at our institution between July 2017 and 2022, in whom surgical decision-making between BVR or UPV could not be decided from preoperative echocardiography alone and therefore 3D-reconstruction and 3D-printing had been performed to support decision-making, were included in this study. Patients were excluded if surgical decision was made from standard preoperative echocardiographic assessment and no additional CT or MRI data was required. Both echocardiography and 3D image data from CT or MRI were analyzed for the included patients. Heart team meetings for determining the treatment decision were only performed with the additional information obtained via 3D reconstruction and 3D printing was available. Reasons for these individual cases to be perceived as complex and in need of additional information for treatment planning are presented in the results section.

Preoperative parameters such as age, weight, anatomical characteristics or previous catheter and surgical interventions were recorded. Additionally, intraoperative characteristics, such as type of procedure, total operating time, cardiopulmonary bypass time, intraoperative complications, and postoperative characteristics, such as total ventilation time, total intensive care unit stay, total hospital stay, postoperative complications, were obtained.

### Preoperative echocardiographic measurements

Echocardiographic data concerning chamber sizes, AV-valve sizes and VSD morphology of included patients was retrospectively analyzed offline from digitally archived preoperative echocardiograms with a vendor-specific software (EchoPAC™ version 203; General Electric Vingmed Ultrasound AS, Horten, Norway). Measurements were performed according to current recommendations (18). For available parameters, Z-Scores were calculated (19, 20).

### 3D image data

The image data available for 3D reconstruction was heterogenous in quality. In eight patients a CT scan had been performed using a Siemens Somatom Definition Flash scanner (Siemens Healthcare GmbH, Erlangen, Germany). Tube currents were adapted individually according to the body mass (CARE Dose), whereas a constant tube voltage of 100 kV was used (CARE kV). Spatial resolution of the CT images varied from 0.27 × 0.27 mm^2^ to 0.40 × 0.40 mm^2^, based on the slice thickness of 0.50–0.70 mm and reconstruction increment of 0.40 mm. While the CT device used has a minimal slice thickness of 0.625 mm, the lower values result from overlapping acquisition and respective reconstruction of the image data. Image acquisition was not ECG gated to reduce applied radiation doses. For one patient, cardiac MRI had been performed using a 1.5 Tesla Philips Achieva scanner (Philips Medical Systems, Best, Netherlands) with a 5-element cardiac phased-array coil. For assessment of the end-diastolic anatomy, a balanced 3D steady-state-free-precession imaging sequence (3 signal averages, navigator gated, ECG triggered) was used.

### 3D image reconstruction

**FIGURE 1:**
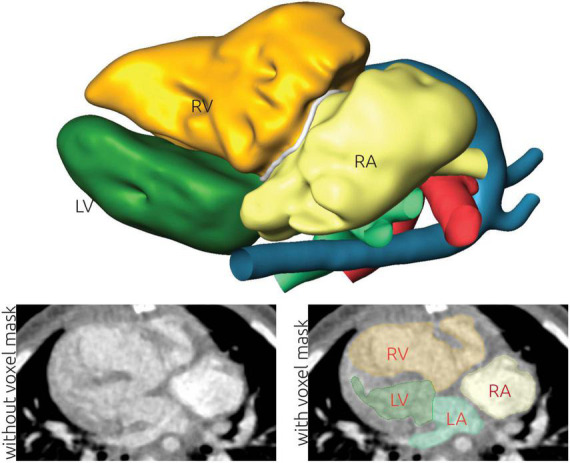
Illustration of the image-based reconstruction of a patient-specific anatomy using computed tomography (CT) data. The top panel shows the resulting 3D surface geometry of the patient. The lower left panel shows a CT image slice without labels. The grayscale window is set to a range from -500 to +500 Hounsfield units. This image is superimposed using the labels generated during image reconstruction in the lower right panel. Here, the left (LA) and right atrium (RA), as well as the left (LV) and right ventricle (RV) are highlighted.

Reconstruction of the patient-specific anatomy from both CT and MR image data was performed using AmiraZIBEdition (v. 2022.3; Konrad-Zuse-Zentrum Berlin, Germany). The aim of this procedure was to label all image voxels belonging to the blood pool. No reconstruction of the myocardium was performed. Entirely manual tools as brushes, as well as semi-automatic tools as region growing algorithms were employed. Due to the heterogeneous and complex anatomies of the individual patients, automatic image reconstruction was not viable. Furthermore, the heterogenous image data as well as the uneven distribution of contrast agent in the heart chambers did not allow for specification of a standardized Hounsfield unit threshold to be used for all CT data sets. In general, lower thresholds between 80 and 200 Hounsfield units were used to identify voxels belonging to the blood pool. Distinct labels were generated for the left and right ventricle, the left and right atrium, as well as the aorta and pulmonary artery (see Figure 1). The labeling procedure was performed by progressing through the 3D image stacks slice by slice using axial, sagittal and coronal orientations. Each orientation had to be processed multiple times to correctly identify all relevant anatomical structures. If discernable, neither trabeculae nor papillary muscles of the left and right ventricle were included in the respective reconstructions. All reconstructions were performed by one engineering researcher with more than 10 years of experience in image reconstruction of various pathologies. After an initial draft, each reconstruction was discussed and evaluated with pediatric cardiac surgeons and corrected if necessary.

### 3D model generation and anatomical measurements

From the final voxel mask, a rough initial surface was generated using a marching cubes algorithm implemented in AmiraZIBEdition. This initial surface was subsequently smoothed using a volume preserving smoothing algorithm implemented in JavaView (v.5.01) (21). Subsequently, the smoothed surface was imported into Meshmixer (v3.5, Autodesk, USA), where remaining topological errors were corrected. Most of these errors resulted from artificial connections between anatomically non-connected structures due to proximity of the respective voxel fields, as for example between the aorta and pulmonary artery. Finally, the separation of the different parts of the anatomy was realized on this surface geometry by manually selecting the saddle points indicating the mitral and tricuspid annulus, the perimeter of the VSD as well as the pulmonary and aortic annulus. While the voxel label field already contained separate labels for the different heart chambers, the limitations of the three cartesian orientations did not allow to correctly model the complex anatomy especially observed in the annuli of the atria-ventricular valves as well as the VSD. Finally, a separate surface geometry for the LV and RV, the left and right atrium, the aorta, the pulmonary artery, as well as all four valvular annulus planes and the VSD were stored as triangulated surface mesh. An example of the final surface reconstruction of one geometry together with the labeled CT images is presented in Figure 1. Different visualization strategies were identified for each patient together with pediatric cardiac surgeons. This *a priori* specification of visualizations was necessary to account for the short schedule available for each patient during heart team meetings. Visualization strategies included showing structures in transparent that obstructed relevant anatomical structures or showing cut-open surface geometries of the ventricles to better highlight in the intra-cardiac structures. Exemplary visualizations used during heart team meetings are provided for all patients as Supplementary material.

**FIGURE 2:**
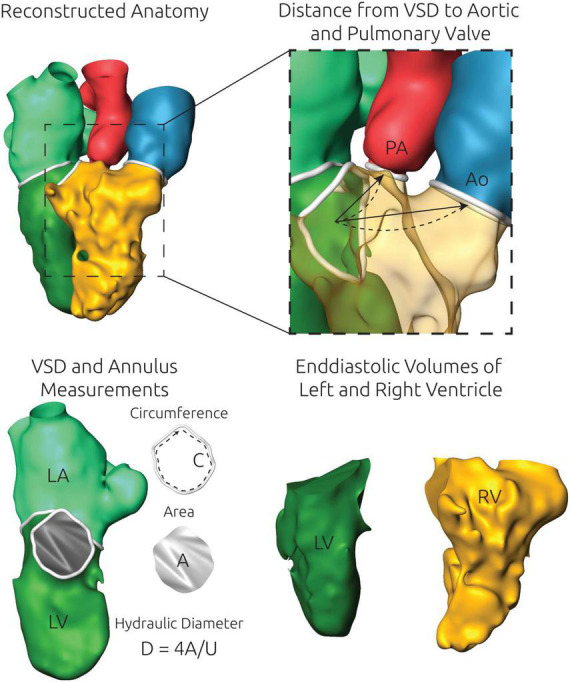
Illustration of the anatomical measurements performed using the specific surface reconstructions of each patient. An exemplary 3D surface geometry is shown in the upper left panel. Top right) The distance from the center of mass of the ventricular septal defect (VSD) and the aortic and pulmonary valve annulus were measured automatically by two approaches. First, the shortest linear connection (arrow) was calculated. Second, a cubic spline (dashed line) was generated to calculate a more realistic curved path. Bottom left) For each valve annulus as well as the VSD, which is shown here exemplarily, the circumference C and the cross-sectional area A were measured. From those two measurements the hydraulic diameter D was calculated. Bottom right) The left (LV) and the right ventricle (RV) were separated from each other and the exact volume of the closed surface geometries of both structures was measured.

Using these reconstructed surfaces, several anatomical parameters were measured for each patient. These include the volume of the reconstructed LV and RV. Additional length measurements were obtained for the RV, by using a cutting plane mimicking the four-chamber view. Then, the maximum distance from the tricuspid annulus to the RV tip was measured within this plane. For each annulus plane as well as the VSD, the cross-sectional area A, the circumference C, and the hydraulic diameter (D = 4*A/C) were calculated. The distances from the VSD to the aortic as well as pulmonary valve annulus were calculated in two ways. First, the distance from the centers of mass of the VSD and the respective valve was calculated. Second, a cubic spline demonstrating the “route” was generated, connecting the centers of mass of the VSD and the respective valve annulus. Here, an additional point was generated halfway between both centers of mass. The position of this center point was manipulated manually, using AmiraZIBEdition, to ensure that the resulting spline lies within the reconstructed blood pool. These two distance measurements were calculated as an estimate of the required length of a tunnel reconstruction via a patch insertion (see Figure 2).

### 3D printing of selected models

Physical models of the patient-specific anatomy were 3D printed using either a Form 2 or a Form 3 (Formlabs, Massachusetts, USA) printer. Here, the surface geometry of the patient-specific blood pool was artificially extruded by a thickness of one millimeter using the extrude command in Meshmixer. Thus, a thin-shelled representation of the blood pool was reconstructed that aimed at mimicking the conventional view a surgeon will have during the procedure, by providing a positive model rather than the negative blood pool model. These models were then cut at a plane or contour identified together with a cardiac surgeon, resulting in two or more pieces allowing the assessment of intracardiac anatomy (the detailed procedure for preparing the 3D prints is provided as Supplementary material). All models were printed using rigid resin [Formlabs Clear Resin RS-F2-GPCL-04 or Gray Resin RS-F2-GPGR-04, Somerville (MA), USA], resulting in a stiff non-pliable model. This approach does not represent the patient-specific myocardium and is thus, not suited for assessment of tissue thicknesses. Two additional models were printed via selective laser sintering of polyamide using a commercial print-on-demand service.

### Statistical analysis

Baseline, intraoperative and outcome measures for patients undergoing UVP vs. BVR are presented as median [interquartile range (IQR)] when reporting continuous data or as frequency (%) when reporting categorical data. Due to the small sample size, only descriptive statistics are given and no comparative analyses of patients undergoing UVP vs. BVR were performed. For the anatomical parameters that were calculated based on the surface reconstructions, average values for both subgroups as well as the entire sample were calculated. To compare echocardiographic measurements and measurements based on the 3D reconstructions, Pearson correlation was calculated for each parameter that was available from both approaches. This analysis was performed using IBM-SPSS version 24.0 (IBM-SPSS Inc, Armonk, NY).

## Results

### Patient characteristics

A total of 64 DORV patients were operated at our institution in the study period between 2017 and 2022; 18 patients received UVP, 43 BVR, one patient underwent 1 1/2 correction and two patients with interim palliations await intended BVR. Nine out of these 64 DORV patients, had preoperative 3D imaging performed since the surgical approach (BVR vs. UVP) could not be conclusively decided based on preoperative echocardiography and were included in this retrospective study. Median age within the study cohort was 13.2 months (IQR: 9.6–24.0) and median weight was 8.3 kg (IQR: 7.8–11.0). No genetic disorders or preterm births were present. Cardiac anatomy was complex among all patients with multiple additional cardiac malformations (see Table 1 for further details). The DORV-VSD subtype was found in three patients, six patients had a DORV-TGA morphology. Four patients had significant pulmonary valve stenosis/RVOTO prior to surgery, the remaining five patients underwent previous pulmonary artery banding before BVR or UVP (Table 1).

**TABLE 1:** Baseline characteristics.

Variable	BVR patients (*N* = 5)	UVP patients (*N* = 4)
Age at surgery (years, median, IQR)	12 (9.6–22.8)	12 (8.7–31.2)
Sex (male, n,%)	4 (80%)	1 (25%)
Weight at surgery (kg, median, IQR)	8.3 (7.4–11.0)	8.8 (7.7–12.9)
BSA (m^2^, median, IQR)	0.4 (0.4–0.5)	0.4 (0.4–0.6)
**DORV type (n,%)**		
DORV-VSD type	2 (40%)	1 (25%)
DORV-TGA type	3 (60%)	3 (75%)
Heterotaxy (n,%)	1 (20%)	1 (25%)
**Associated cardiac malformations (n,%)**		
Right-sided aortic arch	2 (40%)	0 (0%)
Anomalous pulmonary venous return	1 (20%)	0 (0%)
Left superior vena cava	1 (20%)	4 (100%)
Ventricular inversion	0 (0%)	1 (25%)
AVSD	0 (0%)	1 (25%)
Preoperative oxygen saturation (%, median, IQR)	80.0 (76.5–90.5)	77.5 (75.0–83.8)
Prior catheter interventions (n,%)	2 (40%)	1 (25%)
Balloon atrial septostomy	2 (40%)	1 (25%)
Prior surgical interventions (n,%)	4 (80%)	4 (100%)
Pulmonary artery banding	3 (60%)	2 (50%)
Aortopulmonary shunt (central)	0 (0%)	1 (25%)
Stage II. palliation (Glenn procedure)	1 (20%)	0 (0%)

AVSD, Atrioventricular septal defect; BVR, Biventricular repair; DORV, Double outlet right ventricle; IQR, Interquartile range; TGA, Transposition of the great arteries; UVP, Univentricular palliation; VSD, Ventricular septal defect.

### Preoperative echocardiographic data

Echocardiographic measurements are given in Table 2. Overall, the LV was rather small with an average LV end-diastolic volume Z-Score of -2.5 and end-diastolic diameter Z-Score of -3.6. The RV on the other hand was rather large with a RV end-diastolic area Z-Score of 1.7. In two patients, moderate AV-valve regurgitation was observed. Overall AV valve dimensions were normal (average Z-scores from -0.1 to -1.6). No obvious AV valve straddling was observed by echocardiography.

**TABLE 2:** Preoperative echocardiographic characteristics.

	DORV patients with UVP				DORV patients with BVR					Average values		
	1	2	3	4	5	6	7	8	9	All	UVP	BVR
**Ventricular volumes and sizes**												
**Left ventricle**												
LVEDV (ml)	8.0	8.0	7.0	15.0	15.0	7.0	8.0	16.0	14.0	10.9	9.5	12
LVEDV Z-score	–3.3	–2.9	–4.1	–0.9	–1.6	–3.3	–3.0	–2.3	–1.0	–2.5	–2.8	–2.2
LVEDD (cm)	17.0	19.0	19.0	21.0	22.0	17.0	20.0	24.0	22.0	20.1	19	21
LVEDD Z-score	–4.5	–3.1	–4.6	–2.1	–2.2	–3.9	–2.7	–2.5	–1.5	–2.9	–3.6	–2.6
LV area (cm^2^)	3.9	3.6	5.5	5.6	5.1	3.5	3.5	4.1	4.4	4.4	4.7	4.1
LV area Z-score	–2.1	–1.9	–1.7	0.6	–0.7	–2.0	–2.1	–2.7	–0.7	–1.5	–1.3	–1.6
**Right ventricle**												
RV length 4CV (mm)	40.0	35.0	40.0	25.0	39.0	38.0	38.0	43.0	40.0	37.6	35	39.6
RV length Z-score	1.4	0.7	–0.1	–2.7	1.2	1.5	1.5	0.9	2.3	0.8	–0.2	1.5
RV end-diastolic area (cm^2^)	8.0	7.5	8.2	3.9	7.9	6.9	6.2	9.6	7.5	7.3	6.9	7.6
RV end-diastolic area Z-score	2.3	2.6	0.8	–1.1	2.3	2.2	1.5	2.0	2.9	1.7	1.1	2.2
**VSD size**												
Diameter (mm)	13.0	13.0	16.0	12.0	17.0	11.0	22.0	15.0	8.0	14.1	13.5	14.6
**Geometric measurements MV annulus**												
Diameter 4CV (mm)	*	13.0	13.0	14.0	14.0	11.0	13.0	15.0	14.0	13.2	13.0	13.4
Diameter 4CV Z-score	*	–0.8	–2.5	0.1	–1.3	–2.2	–0.8	–1.1	0.2	–1.2	–1.3	–1.1
Diameter PLAX (mm)	*	11.0	12.0	13.0	15.0	10.0	10.0	14.0	14.0	12.8	13	12.6
Diameter PLAX Z-score	*	–2.4	–3.2	–1.0	–0.3	–3.0	–3.0	–1.9	–0.1	–1.6	–1.6	–1.7
**Geometric measurements TV annulus**												
Diameter 4CV (mm)	*	16.0	16.0	10.0	17.0	15.0	17.0	21.0	17.0	16	14.3	17.5
Diameter 4CV Z-score	*	0.6	–1.0	–2.6	0.6	0.1	1.1	1.4	1.4	0.1	–0.9	0.9
Diameter PLAX (mm)	*	16.0	15.0	13.0	15.0	13.0	16.0	23.0	16.0	16.2	15.8	16.6
Diameter PLAX Z-Score	*	0.6	–1.5	–1.0	–0.4	–1.1	0.6	2.4	0.9	0.2	–0.1	0.5

*In this patient AVSD was present and therefore no separate MV/TV annulus measurements could be performed. 4CV, 4-chamber view; BVR, Biventricular repair; DORV, Double outlet right ventricle; LV, Left ventricular; LVEDD, Left ventricular end-diastolic diameter; LVEDV, Left ventricular end-diastolic volume; MV, Mitral valve; PLAX, Parasternal long axis view; RV, Right ventricular; TV, Tricuspid valve; UVP, Univentricular palliation; VSD, Ventricular septal defect.

### Indications for requesting 3D reconstruction and printing

In all study cases, routine clinical information was considered to be insufficient for adequate treatment planning for various reasons. Primarily 3D reconstruction was proposed to assess, whether closure of the VSD to either the aorta or the pulmonary artery would create a potential outflow tract obstruction or an obstruction of the AV-valve inlet. In many cases ventricular volumes measured using echocardiography as well as initial assessment of the size and location of the VSD suggested BVR to be a valid strategy. While echocardiography can sufficiently visualize the structures of interest, namely the pulmonary and aortic valve, the VSD and the AV valve inlet, the geometry and spatial relationships of these structures can often only be apprehended insufficiently.

### Reconstruction of the patient-specific anatomy

**FIGURE 3:**
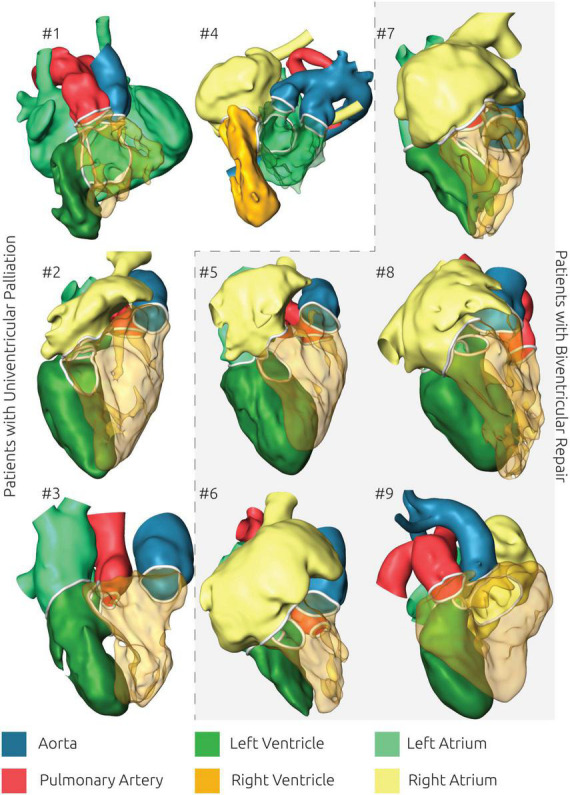
Overview of all reconstructed surface geometries using the same color scheme for highlighting all anatomical structures. Patients with biventricular repair are shown with a gray background.

The patient-specific anatomy of all patients is shown in Figure 3. A rotating animation of the entire anatomy is provided for every patient as Supplementary material. Furthermore, the surface geometries of all regions of the cardiovascular anatomy are also made accessible as Supplementary material. The entire anatomy consisting of the LV/RV, the left and right atrium as well as the aorta and pulmonary artery was reconstructed for all patients except for patient #3, for whom the right atrium was not discernable due to a lack of contrast. For patient #1 the atrial septal wall was not visible in the CT image data so that a joint geometry was generated for the left and right atrium. The required time for image reconstruction varied from 4 to 12 h, depending on the complexity of the anatomy, the image contrast, and the image voxel resolution, as well as the necessary corrections and reviews together with the surgical experts. While most ventricular surface geometries are rather smooth and feature no marked signs of the trabeculae carneae, some patients’ reconstructions feature rather jagged ventricular geometries (e.g., patient #3. #5, #6).

### Qualitative considerations regarding the benefit of 3D reconstructions

One advantage arising from the availability of 3D representations of the patient-specific anatomy is that the spatial orientation of intracardiac structures can be assessed in a qualitative manner as well. Here pediatric cardiac surgeons as well as pediatric cardiologists considered the 3D visualization alone as already being helpful for understanding the usually complex anatomy. Manipulation of this 3D visualization, such as transparent representations of selected parts for example the right ventricle (see Figure 3) as well as the opportunity to freely manipulate the view’s orientation further helped to assess the size, shape and spatial relationship of different intracardiac structures as well as to understand surgical constraints imposed by the patient-specific anatomy.

**FIGURE 4:**
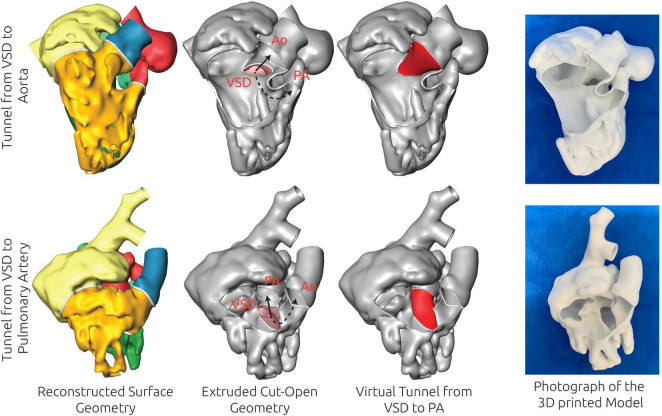
Examples of the virtual treatment decision based on the 3D reconstructions for two patients (patient #7 and patient #8), for which biventricular repair (BVR) was feasible. The 3D reconstruction of the blood pool is shown in the left column, whereas the extruded, thin-walled geometry, superimposed with the relevant structures for treatment planning is shown in the second column. The virtual treatment strategy is shown in the third column, whereas the right column shows a photograph of a 3D print of the model. For patient #8 (upper row) BVR by creation of a tunnel from the ventricular septal defect (VSD) to the aorta (Ao) was considered feasible due to the size and orientation of these structures as well as the size of the left ventricle. In patient #7 the tunnel from VSD to Ao was considered to be too strongly bent and not feasible due to the orientation and shape of the VSD, whereas a connection from VSD to the pulmonary artery (PA) was considered feasible.

Apart from quantitative measures as distances between the VSD and the AV valve annuli, qualitative assessment was considered to allow estimation of potential treatment strategies, such as tunnel orientations, lengths, and sizes. Two examples where the 3D visualization clearly highlighted whether BVR by surgical creation of a tunnel from the VSD toward the aortic valve annulus was feasible are shown in Figure 4. While BVR was possible in both patients, only in patient #8 connection from the VSD to the aortic valve was feasible. In patient #7, this tunnel would have been strongly bent and long due to the constraints imposed by the LV anatomy. In this patient a closure of the VSD toward the pulmonary artery and an additional arterial switch helped creating a straighter and shorter tunnel. The eventual surgical treatment performed in both patients matched these theoretical considerations.

In addition to the 3D visualization, the 3D printed models were considered to offer additional understanding of the anatomy. While the 3D visualization was considered to be better suited to highlight different compartments and intracardiac structures, the 3D printed models were considered to be closer to the real surgical environment, even though the models were rigid, and the myocardium was not reconstructed in a patient-specific manner. A major advantage of the 3D printed models was the intrinsic understanding of the sizes and dimensions of different intracardiac structures. The missing pliability of the models was considered the major constraint, as having soft-tissue models might allow to already mimic different surgical procedures before the intervention.

### Anatomical measurements based on surface reconstruction

The anatomical measurements which were performed on the reconstructed surface geometries are listed in Table 3. Here, all individual measurements as well as the average for all nine patients and the average values for the patients receiving UVP or BVR are provided, respectively. On average, the end-diastolic ventricular volumes were larger in the patients receiving BVR. In general, the RV volumes were larger than the LV volumes in both groups. The distances from the VSD to the pulmonary and aortic valve were similar when averaged for all patients. However, on average for the patients receiving UVP, larger distances to the aortic valve were observed compared to the pulmonary valve. In contrast, the distances between VSD and the two valves were similar in the BVR subgroup. However, similar to the ventricular volumes, these trends were not observed in all individual cases. In patient #1, who received UVP, distances where similar and in patient #5 for whom BVR was achieved, the distance to the aortic valve was larger than that to the pulmonary valve. Size measurement of the heart valves’ annuli and the VSD revealed that the VSD was considerably larger in UVP patients than in BVR patients. Similarly, the aortic annulus was larger in patients with BVR.

**TABLE 3:** Preoperative anatomic features of DORV patients measured using 3D reconstructions.

	Patients with univentricular palliation (UVP)				Patients with biventricular repair (BVR)					Average values		
	1	2	3	4	5	6	7	8	9	All	UVP	BVR
**Ventricular volumes (ml)**												
LV	8.3	15.7	12.9	10.8	27.6	3.4	5.2	20.9	14.2	13.2	11.9	14.3
RV	11.3	29.3	16.9	8.6	29.0	9.2	13.1	22.8	35.7	19.5	16.5	21.9
**Ratio of LV/RV volume** (-)												
LV/RV	0.74	0.54	0.76	1.25	0.95	0.37	0.40	0.92	0.40	0.70	0.82	0.61
**Ventricular length (mm)**												
RV	37.1	37.0	43.7	22.0	45.2	39.2	40.8	47.5	43.2	39.5	35.0	43.2
**Distance between VSD and aortic/pulmonary valve (mm)**												
Aortic valve linear	20.0	34.8	24.4	24.5	28.0	13.8	15.1	13.1	13.3	20.8	25.9	16.7
Aortic valve curved	21.4	33.2	25.6	25.8	29.6	15.2	16.5	15.0	14.8	21.9	26.5	18.2
Pulmonary valve linear	22.8	23.0	11.9	17.4	22.6	13.1	11.4	20.1	15.7	17.5	18.8	16.6
Pulmonary valve curved	25.2	24.7	14.3	19.6	25.2	17.2	13.3	29.4	20.2	21.0	20.9	21.1
**Geometric measurements VSD**												
Area (mm^2^)	303.2	236.1	280.6	123.7	226.0	119.5	235.0	106.8	59.1	189.0	235.9	149.3
Circumference (mm)	67.3	59.5	62.0	40.5	55.1	39.3	64.9	38.3	28.7	50.9	57.3	45.3
Diameter (mm)	18.0	15.9	18.1	12.2	16.4	12.2	14.5	11.2	8.2	14.1	16.0	12.5
**Geometric measurements aortic annulus**												
Area (mm^2^)	84.2	135.3	180.2	147.4	166.9	130.3	159.0	328.8	217.9	172.2	136.8	200.6
Circumference (mm)	36.8	41.9	48.7	45.8	48.4	42.4	45.4	66.4	54.3	47.8	43.3	51.4
Diameter (mm)	9.2	12.9	14.8	12.9	13.8	12.3	14.0	19.8	16.0	14.0	12.4	15.2
**Geometric measurements pulmonary annulus**												
Area (mm^2^)	169.0	110.7	35.6	94.8	170.9	17.9	115.7	92.9	163.5	107.9	102.5	112.2
Circumference (mm)	49.1	38.4	22.9	36.6	47.9	16.1	38.4	36.8	47.1	37.0	36.8	37.3
Diameter (mm)	13.8	11.5	6.2	10.4	14.3	4.4	12.1	10.1	13.9	10.7	10.5	10.9
**Geometric measurements MV annulus**												
Area (mm^2^)	*	130.7	354.8	214.4	262.4	152.8	139.0	270.8	166.9	214.9	235.5	198.4
Circumference (mm)	*	43.4	69.5	54.8	61.2	45.2	42.8	60.3	48.2	54.3	57.8	51.5
Diameter (mm)	*	12.1	20.4	15.6	17.1	13.5	13.0	18.0	13.9	15.4	15.8	15.1
**Geometric measurements TV annulus**												
Area (mm^2^)	*	259.3	*n*.*a*.	181.5	263.4	356.3	316.5	537.7	371.8	328.2	259.9	369.2
Circumference (mm)	*	62.2		50.0	60.8	69.4	82.5	88.9	70.1	70.0	62.8	74.3
Diameter (mm)	*	16.7		14.5	17.3	20.5	15.4	24.2	21.2	18.4	16.3	19.7

*In this patient AVSD was present and therefore no separate MV/TV annulus measurements could be performed. n.a. Data quality did not allow for measurements of the TV. BVR, Biventricular repair; DORV, Double outlet right ventricle; LV, Left ventricular; MV, Mitral valve; RV, Right ventricular; TV, Tricuspid valve; UVP, Univentricular palliation; VSD, Ventricular septal defect.

### Quantitative comparison between 3D-based measurements and echocardiographic assessment of anatomic parameters

Not all parameters that were evaluated via echocardiography were also measured using the 3D reconstructions. The average values of the LVEDV were similar between echocardiography and 3D reconstruction. Also, the trend of slightly larger LV volumes in the BVR group was observed in both approaches. A moderate correlation between both measurements of 0.67 was found. However, absolute volumes of several patients varied between both modalities (Tables 2, 3). For the RV length measurement an excellent correlation of 0.94 was found. The echocardiographic assessment of the VSD diameter revealed slightly larger VSD in patients receiving BVR than in those receiving UVP. However, the VSD diameters measured using the 3D reconstruction were larger in the UVP group. The correlation between both measurements was 0.50. Similar correlations were found for the TV and MV diameters. For the TV, correlation coefficients between echocardiography and 3D reconstruction were 0.70 and 0.59 for the four-chamber and parasternal long axis view, respectively. Those values were 0.36 and 0.40 for the MV respectively.

### Patients’ outcome

Five patients underwent BVR, four UVP. Reasons why BVR was not feasible in all patients are given in Table 4. BVR was performed by creating an intraventricular baffle closing the VSD and connecting the LV to the aorta in three patients (3/5, 60%) and to the pulmonary artery with an additional arterial switch operation in two patients (2/5, 40%). In two patients (2/5, 40%) the existing VSD was enlarged during BVR. Among the four patients with UVP, one patient underwent Stage I palliation (Damus-Kaye-Stansel anastomosis with modified BT-Shunt), two patients received a Glenn procedure (Stage II palliation), and the remaining patient had a Fontan surgery (Stage III palliation).

**TABLE 4:** Decision-making in DORV patients with UVP.

Patient	Reasons for surgical decision
Patient # 1	AVSD, borderline hypoplastic left ventricle, potential intraventricular tunnel interfering with AV valve inlet
Patient # 2	Non-committed VSD, TV chordae interfering with potential intraventricular baffle
Patient # 3	Non-committed VSD, TV chordae interfering with potential intraventricular baffle, borderline hypoplastic left ventricle
Patient # 4	Non-committed VSD, right ventricular hypoplasia with insufficient volume resulting from potential intraventricular baffle creation

AV, Atrioventricular; AVSD, Atrioventricular septal defect; DORV, Double outlet right ventricle; TV, Tricuspid valve; UVP, Univentricular palliation; VSD, Ventricular septal defect.

## Discussion

DORV represents a heterogenous and complex group of congenital heart defects with considerably variable morphology. Patients frequently require thorough decision-making to ensure optimal treatment tailored for their specific anatomy.

BVR in DORV requires several anatomic prerequisites such as adequately sized ventricles, a VSD that can be rerouted toward either the aorta or the pulmonary artery, the ability to create an adequate outflow tract without compromising ventricular inflow, and a coronary anatomy that allows relocation in case an arterial switch is required. A conclusive decision between BVR vs. UVP is not always straightforward and therefore precise preoperative evaluation with optimal visualization of the anatomy is desired. 3D reconstruction or printing is a rather new technology, which could add additional information to the clinical routine assessment via echocardiography. If anatomy is not comprehended completely, surgical planning, execution and outcomes will be imperfect. Prior studies have demonstrated that improper patient selection for BVR, the generally desired approach, can result in inferior outcomes if anatomic prerequisites are not favorable (6).

The major aim of this study was to focus on this complex group of DORV patients, in which surgical decision-making based on preoperative echocardiography is inconclusive and to evaluate if 3D reconstruction and/or printing can help to better understand anatomy and guide surgical planning. Furthermore, we wanted to access the feasibility of 3D reconstruction and/or printing of the anatomy of DORV patients based on routine image data from CT and MRI and to evaluate whether clinically relevant anatomical measurements could also be performed using these reconstructions.

### Benefits of 3D reconstruction and 3D printing

The overall feedback regarding the additional information provided by either 3D reconstruction and/or 3D printing from the heart team consisting of pediatric cardiac surgeons and pediatric cardiologists was favorable. The possibility of having the entire 3D information of the patient-specific anatomy available was considered beneficial for treatment planning. Furthermore, having 3D printed models allowed *ad hoc* assessment and evaluation of sizes, shapes, relationships, and orientations of various intracardiac structures. Thus, the general feedback from our clinical center matches the experience from other retrospective studies (10–12).

According to members of the heart team, the main advantage provided by both 3D reconstruction and 3D printing was that different treatment strategies could be elaborated and discussed easier, as the entire intra-cardiac anatomy could be analyzed at once. For example, feasibility of a patch insertion to connect the VSD to either the pulmonary artery or the aorta could be assessed directly, as the theoretical path could be easier judged. Combined with the experience of the pediatric surgeons, potential obstructions of the AV valves or long narrow tunnels that would result in outflow tract obstruction could be easier identified when the entire information of the patient-specific anatomy was available. Interestingly, anatomical measurements performed using the 3D reconstructions, were of minor interest, as evaluation of the patency of the ventricular sizes and the size of the atrial septal defect were already considered to be well described by echocardiographic measurements. The main advantage was clearly seen in the qualitative information provided by either 3D reconstruction or 3D printed models, such as position, shape, and orientation of intracardiac structures.

In theory, this information about the patient-specific anatomy is already provided entirely by the 3D reconstructions. Furthermore, these simultaneously allow for strong versatility with respect to the choice of visualization, as for example in highlighting different anatomical structures, making structures transparent or being able to freely zoom and rotate the models. However, the feedback from members of the heart team with respect to the 3D printed models were very favorable of that approach, as it allows easy manipulation of the view, and the anatomy of the heart could be assessed *ad hoc* by touching and rotating the model freely. While the printed models still deviate from the real surgical situation, they were considered closer to this than the visualization on the computer screen. Furthermore, as the models were printed in their actual size, intra-cardiac structures were true to size, even though these sizes might not reflect the end-diastolic state perfectly A major advantage of the 3D printed models was, that they allowed to better describe and discuss treatment strategies by indicating them in the model.

However, 3D printing comes with an additional preparation step, as the models must be pre-processed and printed. Furthermore, depending on the complexity of the model, as well of the printing technology used, post-processing of the models can also require substantial time. In contrast, preparation of the visualizations of the purely digital 3D reconstructions also takes time and was not sufficiently interactive. This problem, however, might be overcome with training or dedicated software solutions allowing *ad hoc* changes of the visualization during the heart team meetings. Finally, common feedback was, that pliable models allowing deformation and probably even mock interventions would be favorable over rigid models.

### Comparison of echocardiographic and reconstruction-based measurements

We also attempted to compare anatomical measurements performed on the reconstructions against echocardiographic measurements performed in clinical routine to assess whether 3D reconstructions could also be used for robust assessment of these parameters. While average measurements available from echocardiography and 3D reconstructions were reasonably comparable, individual measurements showed considerable variability. In particular the VSD diameter was measured substantially different with smaller diameters in UVP patients based on echocardiographic assessment and larger diameters in that the BVR group based on the 3D reconstructions. However, even though several average measurements agreed well, correlations between both modalities were not excellent. Only for the RV length a strong correlation was observed, whereas for all other parameters, correlations ranged between 0.36 and 0.70. One possible explanation for this is that the RV length measurement is defined in the four-chamber view, which was reproduced in the 3D reconstruction for this measurement. Thus, similarity of the evaluation planes could be achieved. Planar echocardiographic measurements are known to be less precise than volumetric measurements (22) and measurements in neonates are known to be operator dependent (23).

Quantifications of cardiac chamber volumes have important inherent limitations in both modalities. Since our CT protocols in children are generally not ECG gated due to higher radiation exposition with retrospective ECG gating, acquired 3D images are not reliably end-diastolic and therefore might underestimate end-diastolic volumes. Echocardiography using the modified Simpson’s method for LV volume measurement may also be inaccurate, especially in complex anatomies, due to incongruence of geometric assumptions but also resulting from poor acoustic windows or limited operator experience. Moreover, RV volumes cannot be reliably measured in conventional 2D echocardiography. Nonetheless, as current reference values are based on echocardiographic measurements and echocardiography allows assessment of the end-diastolic state, measurements based on 3D reconstructions are no viable substitute for assessing ventricular volumes yet. ECG-gated acquisition of CT image data might overcome this problem but would require higher radiation dosage. Also, MRI images are performed ECG-gated. However, due to the long acquisition times, MR imaging usually requires sedation of neonates to prevent motion artifacts and the image resolution is usually poorer than that of CT images.

### Outlook

While the main benefit of 3D reconstruction and 3D printing was seen in providing a clear visualization of the entire cardiac anatomy at once and reconstruction-based measurements were of limited importance, these measurements could be beneficial in future. First, echocardiographic measurements are often performed in 2D representations. In this study, the morphology of the ventricles and VSDs was very complex in several patients. For example, the RV length measurement in the four-chamber view was not ideal to assess the maximum elongation of the ventricle. Furthermore, some VSD shapes featured marked ellipticity, which cannot be assessed using only one diameter measurement. In contrast, the 3D reconstruction provided the exact representation of all anatomical structures, allowing detailed measurements. If the 3D reconstruction procedure can be automated or at least standardized, this approach might allow anatomic quantification without operator biases. Furthermore, additional quantitative measures describing the complex anatomy, such as the orientation of intracardiac structures as well as the orientation of structures in relation to each other might be feasible. Here, parameters such as the angles between the valve annulus planes and the VSD, but also more complex and abstract measurements based on statistical shape analysis (24) can be thought of. Such parameters might allow to objectify the mostly qualitative nature of the additional information provided by either 3D reconstructions or 3D printing.

However, even if such parameters can be identified, 3D reconstruction and 3D printing can currently only be considered as an important complementary diagnostic tool, as not all aspects required for pre-operative treatment planning can be fully assessed from the reconstructions alone. For example, for assessment of the presence of straddling AV valve chordae passing through the VSD, additional echocardiographic examination will always be necessary. Also, identification of novel discriminating parameters for DORV cases eligible for BVR requires much larger sample sizes and a prospective study design, which might be the goal of future studies.

### Limitations

Due to the retrospective nature of this study and the small numbers of patients, the benefits arising from 3D reconstruction and 3D printing of the patient-specific anatomy could only be assessed qualitatively. As 3D reconstruction and 3D printing was only performed for complex cases, where no clear treatment decision could be discerned from routine data, comparison of procedural outcomes of the patients investigated in this study against other patients was not possible. While the first studies aiming at quantification of the added benefit of these technologies exist (17, 25), the conduction of comparative studies of surgical success and/or performance parameters, with and without added information by either 3D reconstructions or 3D-printed models, is not trivial. Since there is neither a consensus nor a standardized approach for reconstruction of the patient-specific anatomy, each center has yet to evaluate these procedures individually to generate evidence and build confidence needed for a prospective investigation.

The reconstruction procedure used in this study requires extensive manual interaction of up to several hours. This surely will be a relevant limitation for any translational endeavors of this approach. However, due to the considerable heterogeneity, both in the patient-specific anatomy but also in the image data, this approach can be considered as beneficial in the current stage of research, as it allows to accurately assess the relevant aspects of the anatomy and mitigate imaging artifacts via the experience of the heart team. The advance of machine learning-based algorithms for image processing was already successful in providing automated tools for reconstruction of different anatomical structures (26–28), including the heart (27), albeit mostly for those with normal physiology. For these approaches to be applicable for congenital heart defects, a joint effort by multiple centers is most likely required to provide the necessary case numbers and sufficiently heterogenous image data for the approach to be widely applicable. Such a database would also be extremely helpful in identifying anatomical parameters with predictive capabilities for BVR or the need for UVP.

One limitation with respect to the comparison between echocardiographic and 3D reconstruction-based parameters is, that both methods are subject to operator biases and uncertainties in its current state, making it impossible to discern the ground truth. Here, prospective studies might allow to assess the accuracy of both methods in more detail, as additional information can be acquired during the surgical intervention.

## Conclusion

Image-based 3D reconstruction of the patient-specific intracardiac anatomy provides important additional information supporting decision-making process and surgical planning. While this information might be useful for further objectification of treatment, the approach is not yet commonly used in clinical routine. Here, one problem is the limited number of prospective studies aiming at quantification of the benefits for treatment-planning. Similarly, the question whether relevant measurements can also be performed directly using 3D reconstruction of the patient-specific anatomy and how these measurements compare against current gold-standard methods cannot be answered sufficiently without additional prospective investigations.

## Data availability statement

The datasets presented in this study can be found in online repositories. The names of the repository/repositories and accession number(s) can be found in the article/Supplementary material.

## Ethics statement

The studies involving human participants were reviewed and approved by Ethikkommission der Charité – Universitätsmedizin Berlin. Written informed consent from the participants’ legal guardian/next of kin was not required to participate in this study in accordance with the national legislation and the institutional requirements.

## Author contributions

JB, PK, LG, AS, PM, NS, TK, FB, JP, and VW: conceptualization, investigation, and writing—review and editing. JB, PK, LG, and VW: data curation. JB, PK, and VW: formal analysis. JB, PK, LG, AS, NS, and VW: methodology. LG, PM, TK, FB, JP, and VW: supervision. JB: visualization. JB and VW: statistical analysis, interpretation of results, and writing—original draft. All authors contributed to the article and approved the submitted version.

## Supplementary material

Movies illustrating the 3D geometry of all nine patients in a rotational animation as well as the reconstructed surface geometries, separated for left and right ventricle, left and right atrium, aorta, and pulmonary artery are provided as supplemental material *via* figshare.com (doi: 10.6084/m9.figshare.20465142, https://figshare.com/s/73e82de8b0ef82950dcb).

## Abbreviations

AV: Atrioventricular
BT-shunt: Blalock-Taussig shunt
BVR: Biventricular repair
CT: Computed tomography
3D/2D: Three/Two-dimensional
DORV: Double outlet right ventricle
ECMO: Extracorporeal membrane oxygenation
LV: Left ventricle
MRI: Magnetic resonance imaging
RV/RVOTO: Right ventricle/right ventricular outflow tract obstruction
TGA: Transposition of the great arteries
UVP: Univentricular palliation
VSD: Ventricular septal defect.
