# Supplementary material for: 3D modeling and printing for complex biventricular repair of double outlet right ventricle
Source: Front Cardiovasc Med. 2022 Nov 30;9:1024053. doi: 10.3389/fcvm.2022.1024053 (PMC9748612; doi:10.3389/fcvm.2022.1024053)
Supplement: Supplementary file 1 [file Data_Sheet_1.DOCX]

# Pre-processing of Reconstructed Surface Geometries for 3D printing

In this document, the procedure for pre-processing of the surface geometries for 3D printing is described in detail. This procedure consists of two major steps:

- separation of the entire cardiovascular anatomy into two or more parts to allow
- extrusion of the reconstructed surface to obtain a thin-walled model allowing to assess the lumen of the different cardiovascular structures

All steps for preparation of the models to print were performed using Meshmixer (v3.5, Autodesk, USA).

## 3D Model of the Patient-Specific Anatomy

The prerequisite of the approach described here, is the 3D reconstruction of the patient-specific cardiac blood pool (4 chambers, large vessels), as shown in Figure 1. This geometry is imported into MeshMixer for subsequent processing.

| 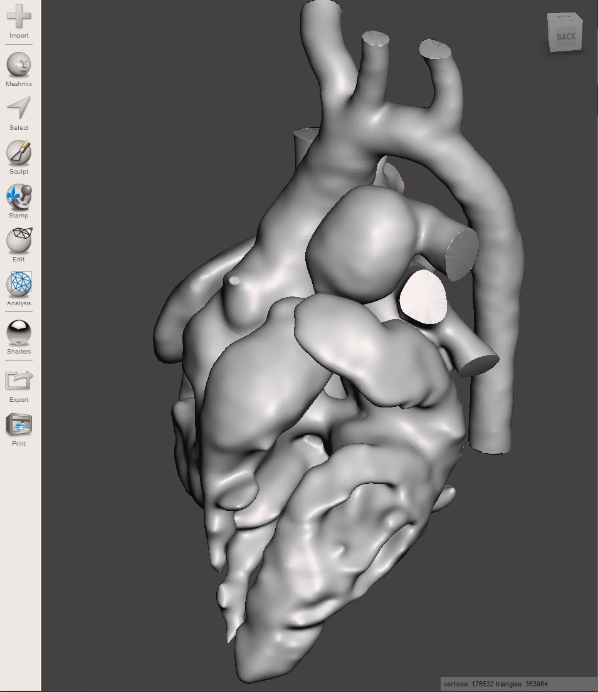Figure A.1: *3D reconstruction of the patient-specific anatomy of the entire heart.* |
| --- |

## Subdivision of the 3D Surface

To allow visual and tactile assessment of the intracardiac structures within the ventricle, the surface geometry must be subdivided into multiple parts. These parts will be extruded in a subsequent step and all parts will be printed separately, allowing to disassemble the heart. In theory, this separation can be performed via planar intersections. However, due to the complex anatomy of the heart, structed that should not be separated might get cut as well. Thus, manual segmentation of the cutting separation of the different parts is recommended.

| 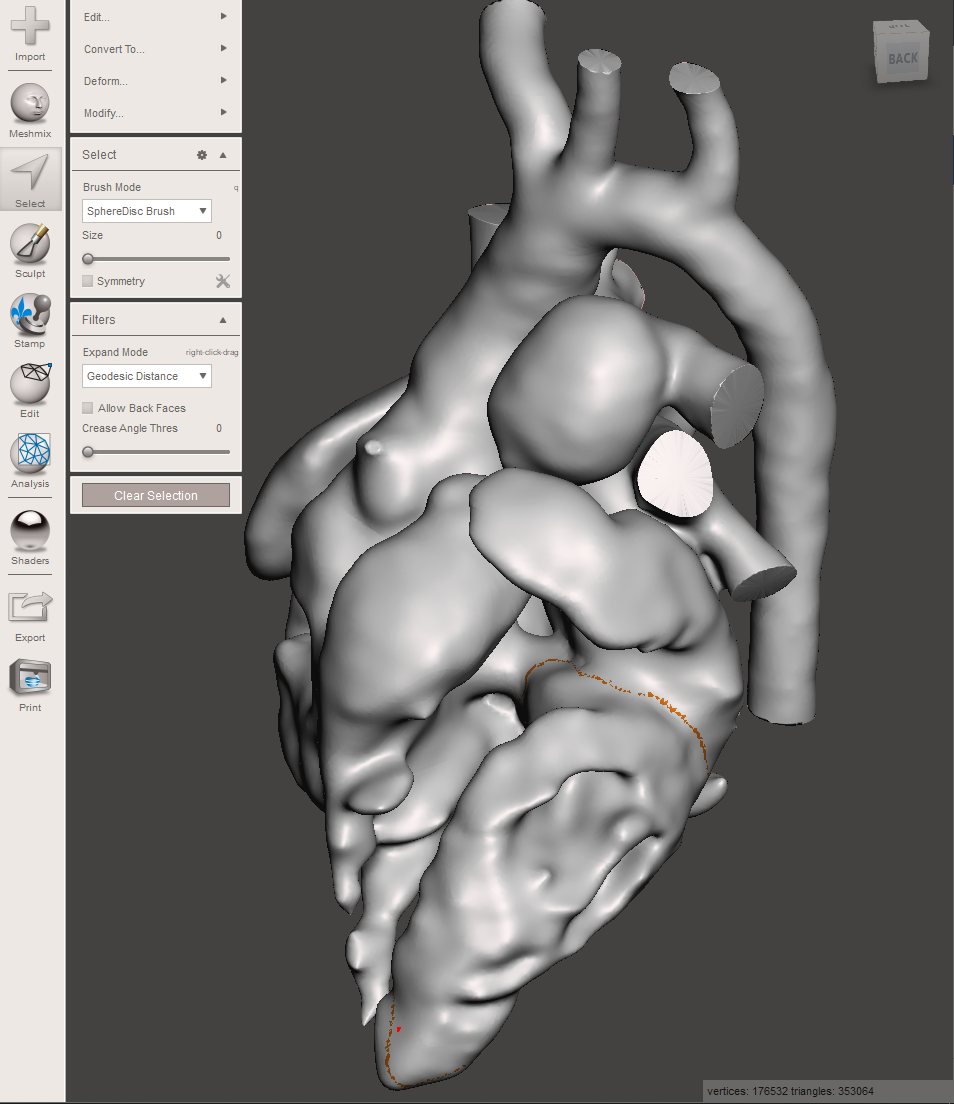 | 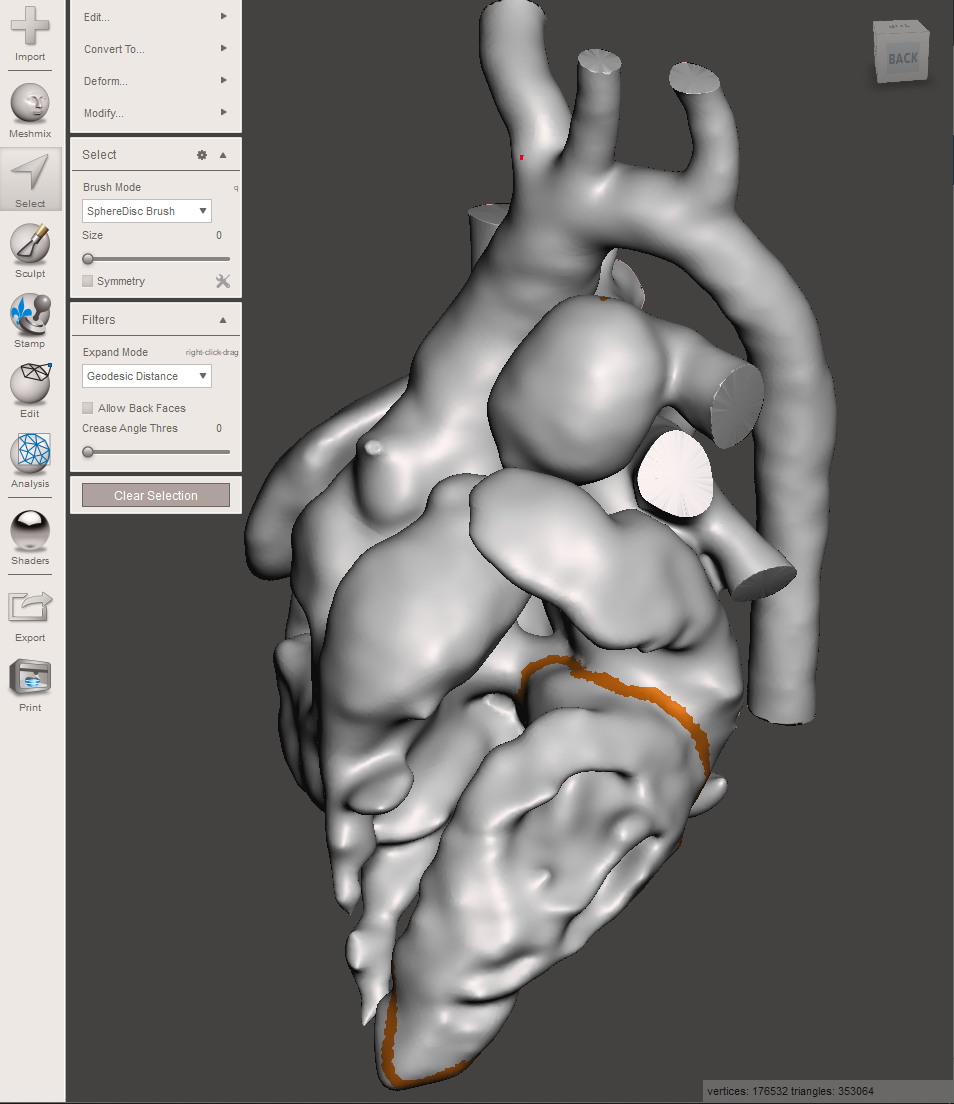 |
| --- | --- |
| 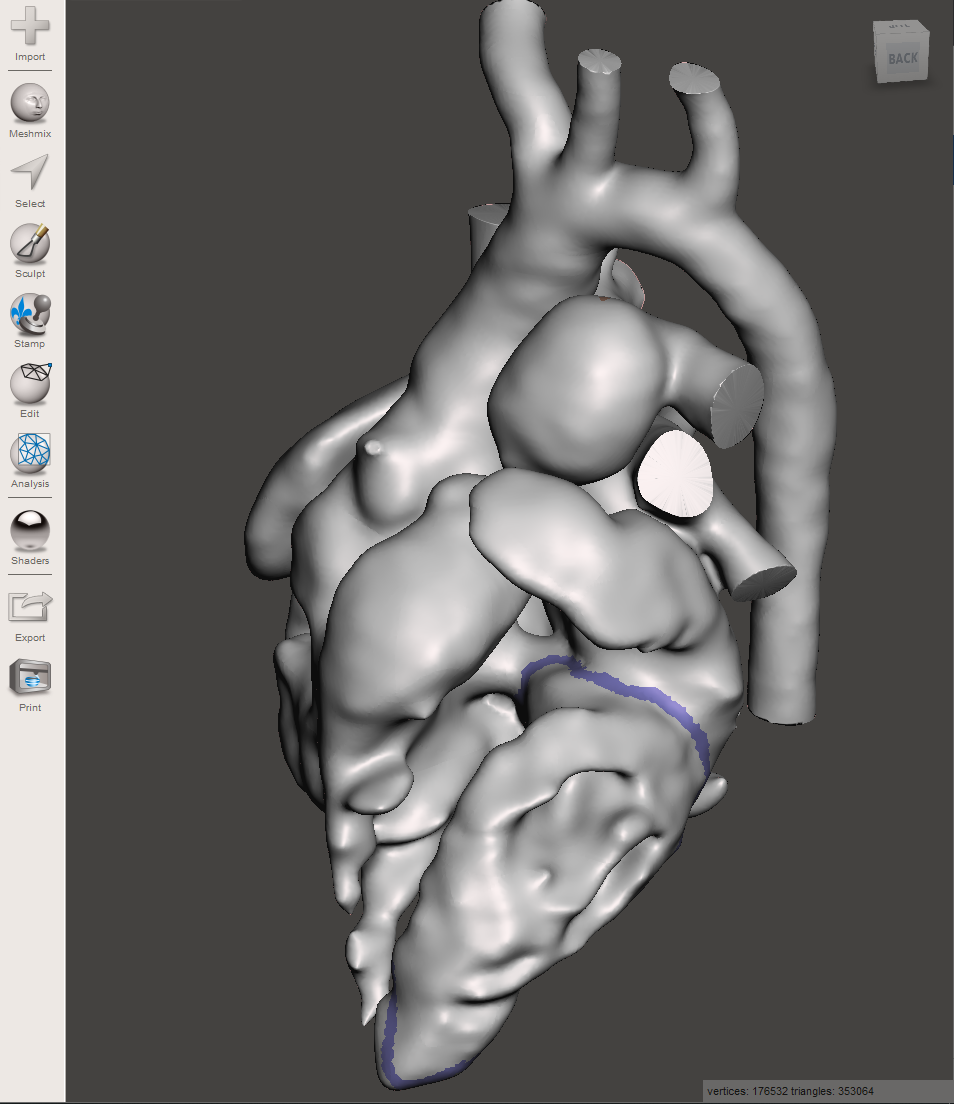 | 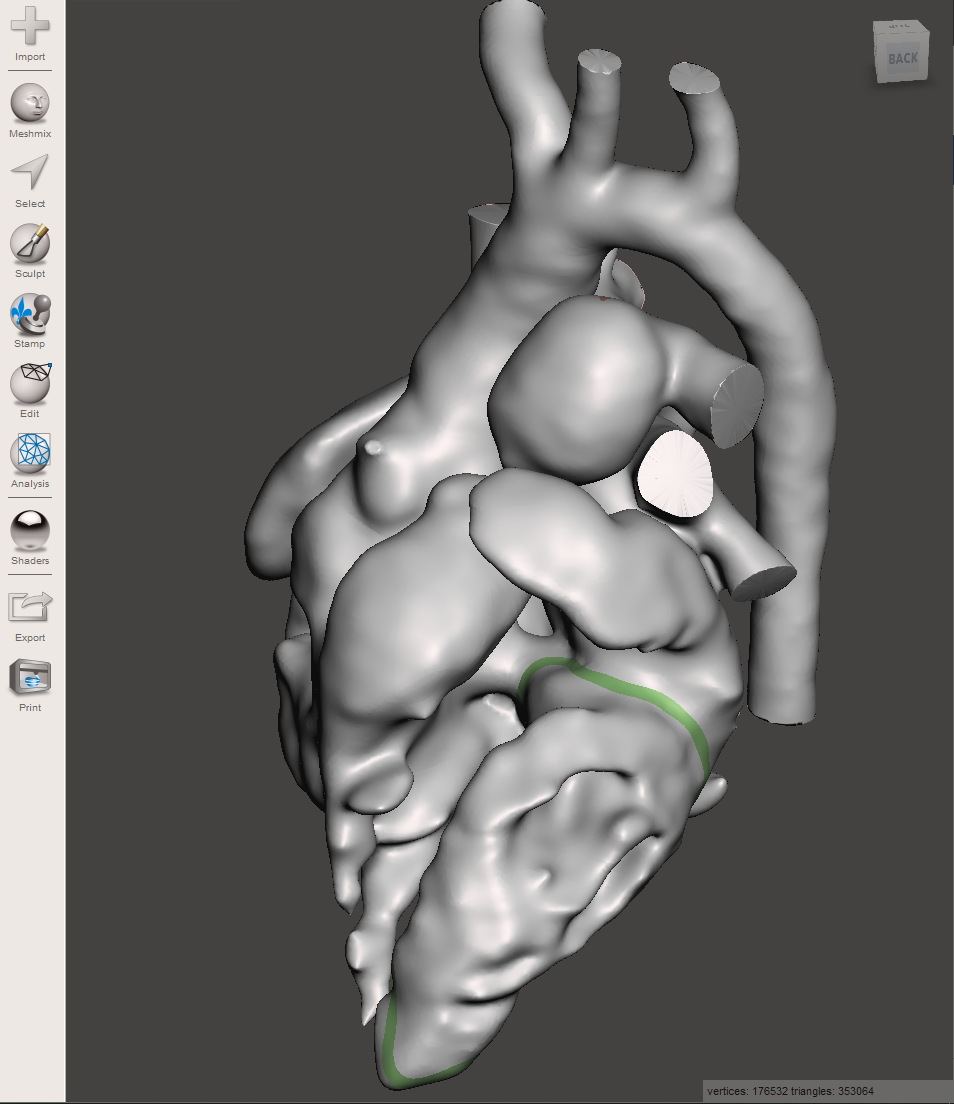 |
| Figure 2: Top left) Manual selection of the contour along which the surfaces are to be separated using the brush tool with the smallest selection size. Top right) Increasing the selection multipe times, to obtain a continous band. Bottom left) Assigning the highlighted faces to their own surface group. Bottom right) Smoothing of the surface group. | |

This procedure is illustrated in Figure 2. First, the selection tool in MeshMixer is used to draw a contour along which the surface is to split. This approach is favorable, as arbitrary contours can be identified, accounting for the specific anatomical constraints. In the shown example, the contour was drawn across the VSD, the mitral valve annuls and the left ventricle aiming to separate only half of the left ventricle from the remaining anatomy. This selection is then increased (shortcut: CTRL * scroll wheel) to obtain a continuous band separating the two regions from each other. This increased selection is then assigned to a new surface group (shortcut: CTRL + G). Due to the triangulation of the surface geometry, the edges of this surface group are jagged. To facilitate a good fit of the 3D printed parts, the contour should be smoothed. To facilitate this, the band is selected again, and the *Smooth Boundary* option is selected (shortcut: B). The option *Preserver Group Borders* has to be deactivated.

## Surface Extrusion

| 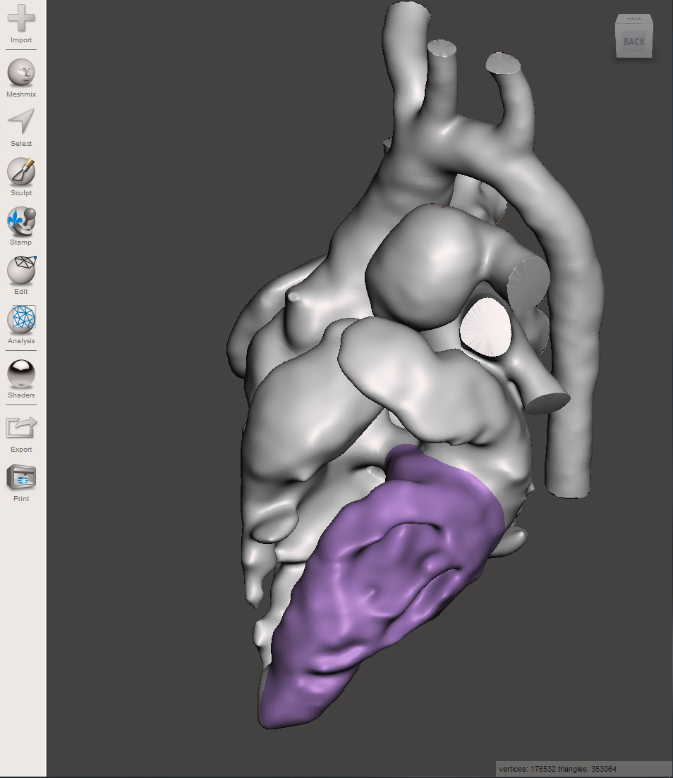 | 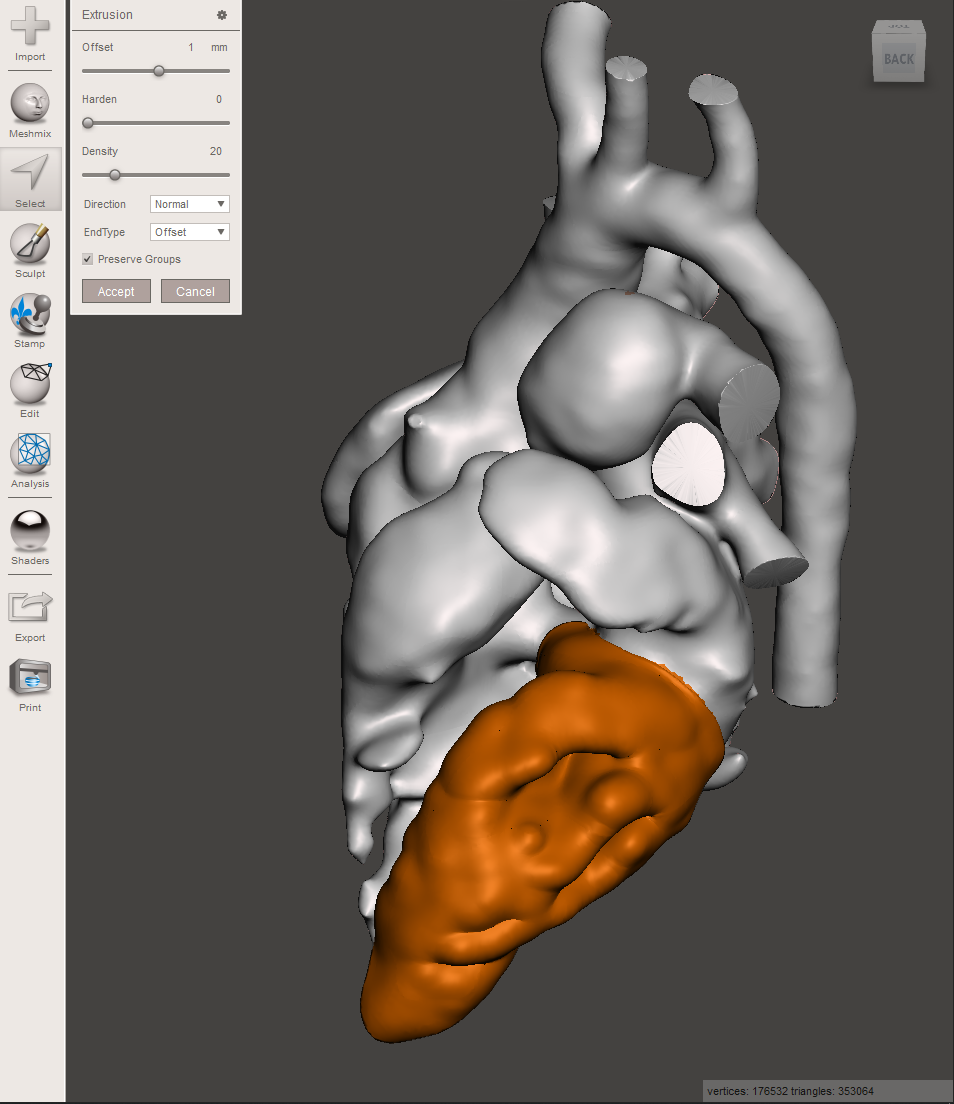 |
| --- | --- |
| Figure 3: *Left) Selection of the left ventricular cap that was isolated in the previous step as well as the band used for separation and assigning both to a joint surface group. Right) Extrusion of the surface group using a constant thickness of 1 mm.* | |

The next step for preparation of the models to be printed is to extrude the surfaces to obtain a thin-walled shell-model of the blood pool. This procedure is illustrated in Figure 3. The left ventricular part that was separated is selected together with the band defined for separation and assigned to a joint group (shortcut: CTRL + G). This group is then selected and extruded (shortcut: D). In the *Extrusion* tool, the thickness can be selected by the *Offset* value. In this example 1 mm was used as wall thickness. The *Direction* of the extrusion has to be set to *Normal*, to ensure that the extrusion is performed using the surface’s normal direction. This step has to be repeated for the remaining parts.

After this step, the different parts can be exported as separate surface files, using file formats such as STL or OBJ, which are commonly used by most slicing software for 3D printing. As the 3D printing procedure heavily depends on the printer technology and software used, this step is not described here. However, the surface geometries generated using this approach can for example be easily printed using most print-on demand services offering selective laser sintering prints, as these do not require any support structure. The final geometry of the previous examples is shown in Figure 4.

| 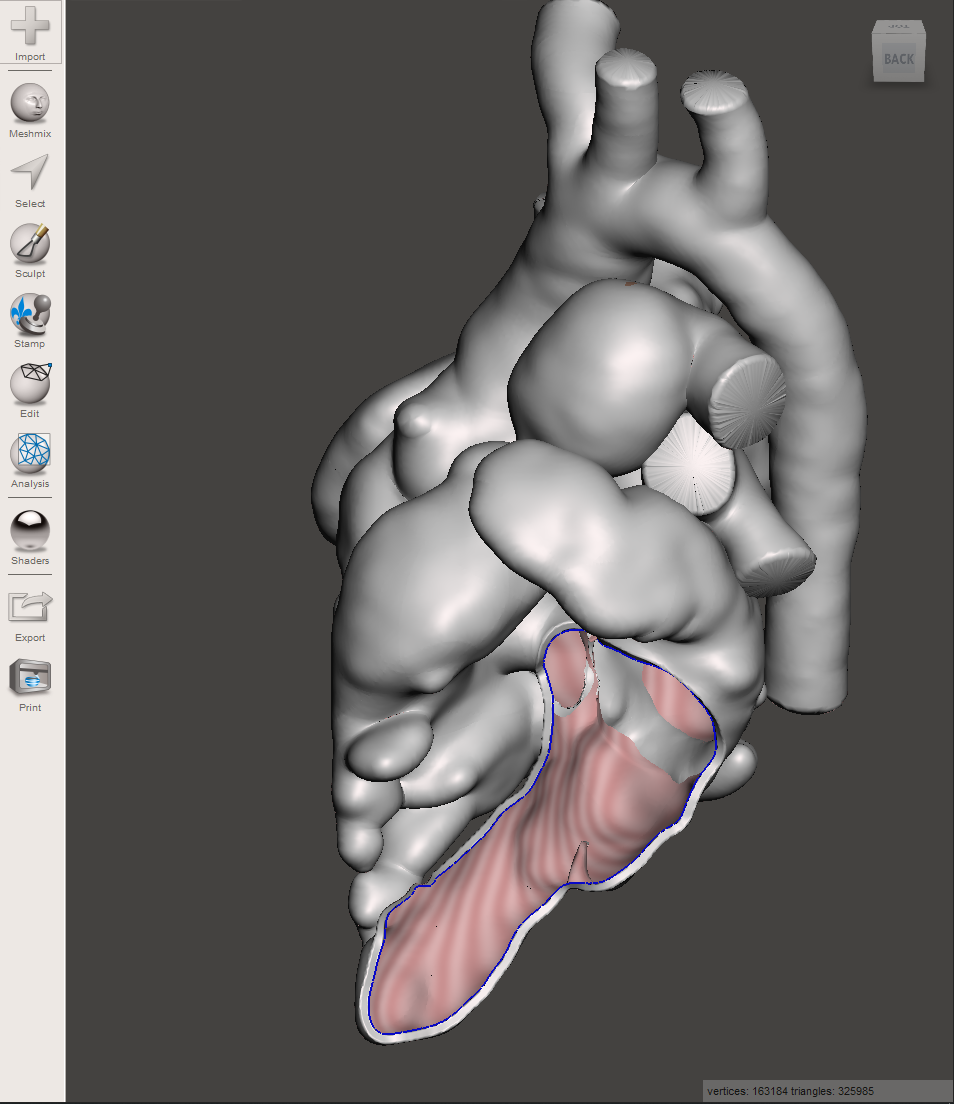Figure 4: *Thin-walled shell of the surface geometry ready for 3D printing.* |
| --- |

## 
